# Developmentally regulated internal transcription initiation during meiosis in budding yeast

**DOI:** 10.1371/journal.pone.0188001

**Published:** 2017-11-14

**Authors:** Sai Zhou, Rolf Sternglanz, Aaron M. Neiman

**Affiliations:** 1 Department of Biochemistry and Cell Biology, Stony Brook University, Stony Brook, NY, United States of America; 2 Graduate Program in Genetics, Stony Brook University, Stony Brook, NY, United States of America; Florida State University, UNITED STATES

## Abstract

Sporulation of budding yeast is a developmental process in which cells undergo meiosis to generate stress-resistant progeny. The dynamic nature of the budding yeast meiotic transcriptome has been well established by a number of genome-wide studies. Here we develop an analysis pipeline to systematically identify novel transcription start sites that reside internal to a gene. Application of this pipeline to data from a synchronized meiotic time course reveals over 40 genes that display specific internal initiations in mid-sporulation. Consistent with the time of induction, motif analysis on upstream sequences of these internal transcription start sites reveals a significant enrichment for the binding site of Ndt80, the transcriptional activator of middle sporulation genes. Further examination of one gene, *MRK1*, demonstrates the Ndt80 binding site is necessary for internal initiation and results in the expression of an N-terminally truncated protein isoform. When the *MRK1* paralog *RIM11* is downregulated, the *MRK1* internal transcript promotes efficient sporulation, indicating functional significance of the internal initiation. Our findings suggest internal transcriptional initiation to be a dynamic, regulated process with potential functional impacts on development.

## Introduction

The generation of gametes through meiosis is the foundation for sexual reproduction in eukaryotes. During this process, diploid cells generate haploid gametes by undergoing one round of DNA replication followed by two rounds of chromosome segregation, namely meiosis I and II. These germ cells later undergo fertilization to restore diploidy, completing the cycle of sexual reproduction. In budding yeast *Saccharomyces cerevisiae*, gametogenesis (also called sporulation) involves the exit of cells from mitotic growth, followed by meiosis and spore morphogenesis, producing four haploid spores from one diploid mother. The coordination between these events during sporulation requires precise transcriptional and translational control of hundreds of genes [1–3].

Initiation of sporulation requires convergence of nutritional and mating type signals at the promoter of the master inducer, *IME1* [4]. Ime1 complexes with the DNA-binding protein Ume6 to activate the expression of early genes through a conserved site named Upstream Regulatory Sequence 1 (URS1) in the upstream regions of these genes [5]. The expression of early genes is essential for pre-meiotic DNA replication, chromosome remodeling and homologous recombination [4, 6]. One of the Ime1/Ume6 complex targets, *NDT80*, encodes a transcription factor which plays a central role in activating genes required for the meiotic divisions during middle sporulation [7, 8]. Ndt80 binds specifically to a site called the middle sporulation element (MSE), which is found in the upstream region of most middle genes as well its own promoter [9]. The induction of *NDT80* and upregulation of middle genes then drives cells to exit the pachytene stage of meiotic prophase and enter the meiotic divisions [7].

Genome-wide approaches like high-resolution tiling arrays and RNA sequencing have revealed unexpected complexity of the transcriptional landscape in eukaryotic cells [10–14]. Global expression profiling found not only splice variants, intergenic and antisense transcripts, but a large number of variants of transcripts with diversified 5’- and 3’- UTRs [15–17]. One common mechanism underlying UTR heterogeneity is alternative transcription start site (TSS) selection, which is prevalent in eukaryotes [10, 15]. Changes in TSS can alter mRNA stability, splicing, and in some cases localization through a variety of mechanisms [18]. At the translational level, variation in mRNA isoforms often leads to changes in translation efficiency [19, 20]. Additionally, although most TSS changes do not influence the coding capacity of the transcript, mRNA isoforms with extended 5'-UTRs often include upstream open reading frames (uORFs) which can function as translational regulators [21]. Alternative TSSs may also occur within the coding region, where the internally initiated transcript can produce a truncated protein isoform with potential implications for the function of the encoded protein. In yeast, for example, alternative TSSs in the *SUC2* gene lead to protein products that do or do not include an ER targeting signal. Thus, one TSS leads to a secreted form of the protein and the other a cytoplasmic form [22, 23]. Alternative initiation of *SUC2* is a normal process but misregulation of initiation can have pathological consequences. In humans, an alternative TSS in an intron of *ALK* creates a truncated protein expressing only the protein kinase domain of the enzyme which promotes tumorigenesis [24]. Thus, misregulated internal initiations can have dramatic functional outcomes like alterations of cell fate.

Sporulation in budding yeast provides a useful system to study alternative TSS selection in a developmental context. Multiple studies of the budding yeast transcriptome during sporulation have documented dynamic changes in transcript architecture including extended 5'-UTRs and intronic TSSs [2, 10, 25]. Additionally, ribosome profiling revealed that many meiosis-specific extended 5’-UTRs include uORFs that may regulate translation of the major reading frame [3]. The 5’-UTR extension of one such gene *SPO24*, which is required for efficient sporulation, is regulated by Ndt80 and enhances the translation efficiency of the main ORF [26]. While hundreds of meiotic genes with extended meiotic 5’UTRs have been described, internal TSSs that might occur during sporulation are less well documented [3, 10].

One documented internal initiation occurs in the *MRK1* gene [2]. After *NDT80* expression, the *MRK1* locus expresses not only a properly spliced full-length transcript, but also an alternative isoform that initiates within the intron and spans the rest of the gene [2]. *MRK1* encodes one of four GSK-3 family serine/threonine protein kinase homologs in budding yeast. The four GSK-3 homologs: *MCK1*, *MRK1*, *RIM11* and *YGK3* share around 70% similarity with human GSK-3β [27]. *MCK1* functions in sporulation to enhance transcription of *IME1*, phosphorylate Ume6, and promote spore maturation as well having important roles in mitotic cells including regulation of cell cycle progression, chromosomal stability and stress signaling [28–34]. *RIM11* is essential for meiotic entry as its product phosphorylates both Ime1 and Ume6 to promote complex formation between the two factors [35–38]. *MRK1* and *YGK3* have been shown to have redundant functions with *MCK1* and/or *RIM11* in various stress responses [34, 39, 40], but except for some redundancy with Mck1 and Rim11 in phosphorylation of Ume6, little is known about the role and regulation of *MRK1* in sporulation [28].

In order to detect sporulation-specific internal initiation sites, we developed an analytic pipeline that enables sensitive, unbiased detection for novel internal TSSs, and applied it to an RNA-Seq dataset from a highly synchronized meiotic time-course. Different genes with internal TSSs are seen in early and mid-sporulation, suggesting that internal initiation is highly dynamic. MSE sites are highly enriched in the upstream regions of mid-sporulation specific internal TSSs, and internal initiation for several of these genes, including *MRK1*, is *NDT80-*dependent. Furthermore, an MSE site within the annotated intron directly regulates *MRK1* internal initiation, which results in the expression of an N-terminally truncated isoform that contributes to sporulation efficiency. These findings suggest that internal initiations are highly regulated and likely represent a common mechanism for gene regulation during development.

## Materials and methods

### Yeast media and strains

Strains used are listed in Table 1. Unless otherwise noted, standard yeast genetic techniques, media and growth conditions were used [41]. *MRK1* was C-terminally tagged with three copies of the HA epitope by PCR-based integration into strain A14201 using the oligos tg-MRK1-F and -R and the plasmid pFA6a-3HA-HisMX6 [42] as template to generate ySZ68. To construct strains ySZ23 and ySZ102, the open reading frame of *MCK1* or *MRK1*, respectively, were deleted by PCR-based gene deletion using the oligos ko-MCK1-F and -R or ko-MRK1-F and -R and the plasmid pFA6a-kanMX6 [42] as template in the haploids AN117-16D and AN117-4B and subsequent mating of the transformants. Two of these transformants, ySZ159 (*mrk1*Δ) and ySZ21 (*mck1*Δ), were crossed and segregants from non-parental ditype tetrads were mated to generate strain ySZ46. Similarly, *RIM11* was deleted from AN117-16D and AN117-4B using oligos ko-RIM11-F and -R and the plasmid pFA6a-kanMX6 to create ySZ18 and ySZ19. A meiotic depletion allele of *RIM11* (*rim11-md*) consisting of *RIM11* under control of the *CLB2* promoter, was constructed by PCR-based gene replacement of 50bp directly upstream of the *RIM11* ATG codon with a P_*CLB2*_*-3HA* cassette in strain AN117-4B using oligos prms-RIM11-F and -R and the plasmid pFA6a-P_*CLB2*_-3HA-kanMX6 as template [43] to create strain ySZ34. ySZ34 was then mated with ySZ18 to obtain ySZ39. ySZ18 and ySZ34 were each mated with ySZ159 and *mrk1*Δ *rim11*Δ or *mrk1*Δ *rim11-md* double mutants, respectively, were obtained by dissection of the diploids. These haploids were crossed to generate ySZ135.

**Table 1:** Yeast strains.

Strain name	Genotype	Reference
A14154	*MAT*a *his3*::*hisG leu2*::*hisG trp1*::*hisG lys2 ho*::*LYS2 GAL-NDT80*::*TRP1 ura3*::P_*GPD1*_*-GAL4(848)*.*ER*::*URA3*	Berchowitz, Gajadhar et al., 2013
A14155	*MAT*α *his3*::*hisG leu2*::*hisG trp1*::*hisG lys2 ho*::*LYS2 GAL-NDT80*::*TRP1 ura3*:: P_*GPD1*_*-GAL4(848)*.*ER*::*URA3*	Berchowitz, Gajadhar et al., 2013
A14201	*MAT*a/*MAT*α *his3*::*hisG/his3*::*hisG leu2*::*hisG/leu2*::*hisG trp1*::*hisG/trp1*::*hisG lys2/lys2 ho*::*LYS2/ho*::*LYS2 GAL-NDT80*::*TRP1/GAL-NDT80*::*TRP1 ura3*:: P_*GPD1*_*-GAL4(848)*.*ER*::*URA3/ura3*:: P_*GPD1*_*-GAL4(848)*.*ER*::*URA3*	Berchowitz, Gajadhar et al., 2013
AN120	*MAT*a*/MAT*α *ura3/ura3 his3*Δ*SK/his3*Δ*SK leu2/leu2 trp1*::*hisG/trp1*::*hisG lys2/lys2 arg4-NSP1/ARG4 ho*Δ::*LYS2/ho*Δ::*LYS2 rme1*Δ::*leu2/RME1*	Neiman, Katz et al., 2000
AN117-16D	*MAT*a *ura3 his3*Δ*SK leu2 trp1*::*hisG lys2 ho*::*LYS2*	Neiman, Katz et al., 2000
AN117-4B	*MAT*α *ura3 his3*Δ*SK leu2 trp1*::*hisG lys2 ho*::*LYS2 arg4-NSP1 rme1*::*LEU2*	Neiman, Katz et al., 2000
AN262	*MAT*a*/MAT*α *ura3/ura3 his3*Δ*SK/his3*Δ*SK leu2/leu2 trp1*Δ::*hisG/trp1*Δ::*hisG lys2/lys2 ARG4/arg4-NspI RME1/rme1*Δ::*LEU2 ho*Δ::*LYS2/ho*Δ::*LYS2 chs3*Δ::*His3MX6/chs3*Δ::*His3MX6*	Coluccio et al., 2004
ySZ18	As AN117-16D, plus *rim11*Δ::*kanMX6*	this study
ySZ19	As AN117-4B, plus *rim11*Δ::*kanMX6*	this study
ySZ21	As AN117-4B, plus *mck1*Δ::*kanMX6*	this study
ySZ23	As AN120, plus *mck1*Δ::*kanMX6/mck1*Δ::*kanMX6*	this study
ySZ34	As AN117-4B, plus P_*CLB2*_*-RIM11*::*kanMX6*	this study
ySZ39	As AN120, plus *rim11*Δ::*kanMX6/* P_*CLB2*_*-RIM11*::*kanMX6*	this study
ySZ44	As A14154, plus *mrk1-mMSE*	this study
ySZ46	As AN120, plus *mrk1*Δ::*kanMX6/mrk1*Δ::*kanMX6 mck1*Δ::*kanMX6/mck1*Δ::*kanMX6*	this study
ySZ48	As AN117-16D, plus *mrk1-mMSE*	this study
ySZ68	As A14201, plus *MRK1-3HA*::*His3MX6/MRK1*	this study
ySZ69	As A14201, plus *mrk1-mMSE/mrk1-mMSE*	this study
ySZ76	As A14155, plus *mrk1-mMSE*	this study
ySZ98	As AN120, plus *mrk1-mMSE/mrk1-mMSE rim11*Δ::*kanMX6/* P_*CLB2*_*-RIM11*::*kanMX6*	this study
ySZ102	As AN120, plus *mrk1*Δ::*kanMX6/mrk1*Δ::*kanMX6*	this study
ySZ135	As AN120, plus *mrk1*Δ::*kanMX6/mrk1*Δ::*kanMX6 rim11*Δ::*kanMX6/* P_*CLB2*_*-RIM11*::*kanMX6*	this study
ySZ159	As AN117-16D, plus *mrk1*Δ::*kanMX6*	this study
ySZ311	As A14155, plus *mrk1-mMSE-3HA*::*His3MX6*	this study
ySZ313	As A14201, plus *mrk1-mMSE-3HA*::*His3MX6/mrk1-mMSE*	this study
ySZ315	As ySZ135, plus *ura3*::*sMRK1*::*URA3/ura3*	this study
ySZ317	As ySZ39, plus *ura3*::*URA3/ura3*	this study
ySZ318	As ySZ135, plus *ura3*::*URA3/ura3*	this study

To construct the *mrk1-mMSE* strains ySZ48, ySZ44 and ySZ76, 9 nucleotides containing an MSE consensus within the *MRK1* intron (CGACACAAA) were replaced with the kanMX6 cassette by PCR mediated gene replacement in AN117-16D, A14154 and A14155 using oligos ko-MRK1_MSE-F and -R and the plasmid pFA6a-kanMX6. CRISPR/Cas9 was then used as described earlier [44] to replace the kanMX6 cassette with the sequence CGGAATTCA, restoring the original length of the intron but mutating the MSE and creating an EcoRI restriction site. Briefly, the strain was co-transformed with the plasmid pRS425-Cas9-kan280 (gift of G. Zhao) and a double stranded rescue oligonucleotide containing the mutated MSE sequence flanked by 35 nucleotides identical to the chromosomal sequences on each side of the wild-type MSE (oligos rp-MRK1_mMSE-F and -R). Correct introduction of the mutated MSE (mMSE) was confirmed by DNA sequencing. ySZ44 and ySZ76 were crossed to generate ySZ69. To create *mrk1-mMSE* tagged with 3xHA on the C-terminus, PCR-based integration was performed using the oligos tg-MRK1-F and -R and the plasmid pFA6a-3HA-HisMX6 [42] as template and transformed into ySZ76 to generate ySZ311. ySZ311 was then crossed with ySZ44 to obtain the diploid ySZ313.

To obtain an *mrk1-mMSE* homozygous diploid in the *rim11-md* background, ySZ19 and ySZ34 were each crossed with ySZ48 and dissected. The double mutants were then screened from the G418 resistant segregants by PCR amplification of the MSE region followed by EcoRI digestion, and the appropriate haploids were mated to generate ySZ98. To construct the integration plasmid carrying only the internally initiated, short version of *MRK1* (*sMRK1*), the second exon of *MRK1* with 534 nucleotides of upstream sequence and 299 nucleotides of downstream sequence was PCR amplified using oligos cl-sMRK1-306-F and -R with genomic SK1 DNA as template, and cloned into the BamHI/XhoI digested integration plasmid pRS306 (ref) via Gibson assembly to generate pKZ116. This plasmid was linearized by digestion with StuI and transformed into ySZ39 to generate ySZ314. pRS306 was linearized the same way and transformed into ySZ39 and ySZ135, to generate strains ySZ317 and ySZ318, respectively. To construct the integration plasmid carrying only the internally initiated, short version of *MRK1* (*sMRK1*), the second exon of *MRK1* with 534 nucleotides of upstream sequence containing the intron and 3’ end of the first exon and 299 nucleotides of downstream sequence was PCR amplified using oligos cl-sMRK1-306-F and -R with genomic SK1 DNA as template, and cloned into the BamHI/XhoI digested integration plasmid pRS306 via Gibson assembly to generate pKZ116. This plasmid was linearized by digestion with StuI and transformed into ySZ135 to generate ySZ315. pRS306 was linearized the same way and transformed into ySZ39 and ySZ135, to generate strains ySZ317 and ySZ318, respectively.

All deletion and tagged alleles were confirmed by PCR, and all point mutation alleles were confirmed by sequencing. For oligo sequences, see Table 2.

**Table 2:** Oligos.

tg-MRK1-F	CCATTGAAGAACGGTTGAAACATTTTGTTTCTGCACCTTCATCGTCTTTGCGGATCCCCGGGTTAATTAA
tg-MRK1-R	ATGTAATAGTAATGATACAATAGACTAAGAAATTTGAAGGATGAGATATAgaattcgagctcgtttaaac
ko-MCK1-F	AATTTTCTTTTTATTTTCCGAAACCCCCACTCCATCACATTCTAGTACATcggatccccgggttaattaa
ko-MCK1-R	TTGTTCATTAAATTTTCCGAGGGGAAAGAGAACAAATTAATAGAAAATTAgaattcgagctcgtttaaac
ko-MRK1-F	TTGAAAATAATCTCAAGATTGAAGAGGTGAAAATTGTAAAATAGTCTACTcggatccccgggttaattaa
ko-MRK1-R	TGTAATAGTAATGATACAATAGACTAAGAAATTTGAAGGATGAGATATATgaattcgagctcgtttaaac
ko-RIM11-F	TACATTACAACACGGCAACACTAATCACGCAACGCTAGAACTCGCGCAGGcggatccccgggttaattaa
ko-RIM11-R	GTTCCTTCCTTCTCCCATTATTCTTGCCTGGGCTCCCTCCGGTGCTATCAgaattcgagctcgtttaaac
prms-RIM11-F	ACCACCGACGTCTGATTCACACATTACTGGGCAAGATCTTGACATAGCATGAATTCGAGCTCGTTTAAAC
prms-RIM11-R	TTTGACACTATGTTATTACTGAGATTCGGAGAATTATTGCTTTGAATATTCATCTATAAGATCAATGAAGAGAGAGAGGG
ko-MRK1_MSE-F	TTCGTCACAACCGGAGTGAATAGTGGAACTACTGTCAGTTCATAGTATCGcggatccccgggttaattaa
ko-MRK1_MSE-R	TCATAAATCTTTATTTTACACTCTCTTTTTTTTTCTTTTAATTATTTTTTgaattcgagctcgtttaaac
rp-MRK1_mMSE-F	GTGAATAGTGGAACTACTGTCAGTTCATAGTATCGCGGAATTCAAAAAAATAATTAAAAGAAAAAAAAAGAGAGTGTAA
rp-MRK1_mMSE-R	TTACACTCTCTTTTTTTTTCTTTTAATTATTTTTTTGAATTCCGCGATACTATGAACTGACAGTAGTTCCACTATTCAC
cl-sMRK1-306-F	GCGGCCGCTCTAGAACTAGTGGATCCGTACCTAATTTCAATAACAGTTCA
cl-sMRK1-306-R	AGCTGGGTACCGGGCCCCCCCTCGAGATCGCAAGTTTCATCAATTTTTCT

### Sporulation conditions

To sporulate strains containing the estradiol-inducible version of *NDT80* [45, 46], cells were grown in yeast extract-peptone-dextrose (YPD) medium overnight at 30°C until saturation, then diluted in yeast extract-peptone-potassium acetate (YPA) medium to an optical density at 660nm (OD_660_) of 0.25. After incubation at 30°C until the culture reached an OD_660_ of ~1.2, cells were centrifuged at 3,000 x g for 2 min, washed with dH_2_O, and resuspended in sporulation medium (SPO, 2% potassium acetate) at a final OD_660_ of 1.6. The cells were incubated in SPO for 6 h to allow for entry into meiosis and arrest in meiotic prophase and then β-estradiol was added to a final concentration of 1 μM to induce *NDT80* expression. For assays of sporulation efficiency, ascal type distribution and ether sensitivity in strains carrying native *NDT80*, sporulation conditions were similar except there was no addition of β-estradiol, and for sporulation at 16°C, cells were cultured in a refrigerated shaker after shift to SPO. After incubation in SPO medium (> 48 h for cells at 30°C; > 96 h for cells at 16°C), cells were fixed in 3.7% formaldehyde and analyzed under a Nikon Eclipse E200 microscope.

### Computational analysis of RNA-seq data for internal TSS identification

RNA-Seq data obtained from a highly synchronized yeast meiotic culture (GEO database: GSE90008) was used for the computational analyses. In this culture, cells carrying copper-inducible forms of *IME1* and *IME4* were incubated in SPO medium for two hours before copper was added to induce sporulation (GEO database: GSE90008; [47]). Data of various time-points from this time-course dataset was analyzed in this study: the early-meiotic samples refer to samples taken 0.25 h, 0.5 h and 0.75 h after addition of copper (when early meiotic genes are highly expressed); the mid-meiotic samples refer to samples taken 3.75 h, 4h and 4.25 h after induction (when meiotic divisions start); the sample taken at 2 h in SPO medium (when copper was added) was used as the control. The sequencing reads were aligned to yeast reference genome *Saccharomyces cerevisiae* S288C R64.1.1 using TopHat v 2.1.0 [48]. Reads that mapped to the mitochondrial DNA, 2-micron plasmid, rRNA or tRNA genes were removed with BEDTools [49] for accurate quantification, and coverage per nucleotide position was obtained with BEDTools genomecov function.

**Fig 1:**
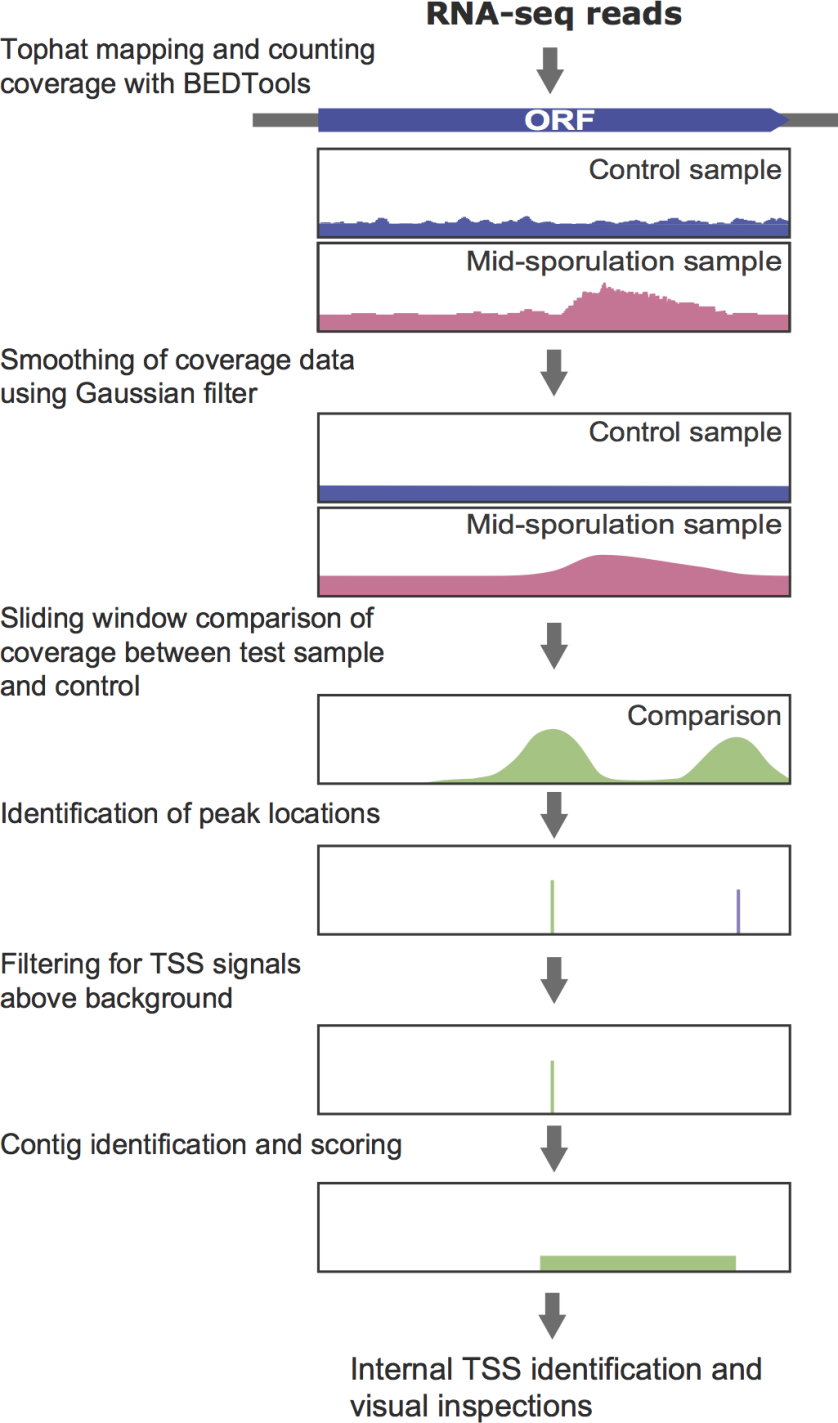
Analytical flowchart for calling mid-sporulation specific internal TSSs from RNA-Seq data of control and mid-sporulation samples. RNA-seq reads of synchronized yeast meiotic culture with P_*CUP1*_-*IME1* P_*CUP1*_-*IME4* strain at *IME1* and *IME4* induction (2 h in SPO) and mid-meiosis (3.75 h, 4 h and 4.25 h after addition of copper) (GSE90008) was used. Analysis flow for a pair of samples is shown as an illustrative example. Reads were aligned to reference assembly (*Saccharomyces cerevisiae* S288C R64.1.1) using TopHat, then quantified as absolute read counts with BEDTools. Coverage data smoothing was performed by the application of a Gaussian kernel on genome-wide read counts. Using sliding windows along the whole genome, the root mean squared deviation between normalized coverage curves of control and test samples reports the shape difference between the curves in each window. Shape difference peaks were filtered to remove potential TES signals (purple line) and signals from noise. For each TSS signal, transcript boundaries were defined into a contig from the difference in smoothed coverage between control and test samples. Close contigs were merged, assigned a score (product of the average of shape difference values and contig size) and ranked. Contigs identified more than once in the analysis of 3 mid-meiotic samples were further evaluated for whether they were internal to known ORFs. Finally, genes with mid-sporulation specific internal TSSs were filtered at an average contig ranking cutoff of 800 and visually examined. Detailed analysis procedures are described in Material and Methods.

For identification of novel meiotic TSSs (TSSs specific to the meiotic sample as compared to the control), a Python script was written for the following analysis (S3 File). First, the coverage data was smoothed and converted to Gaussian kernel density estimates (standard deviation of 50 nt, 99.5% confidence), and normalized to the sum coverage of each sample (referred to as "smoothed coverage" below). The shape of the smoothed coverage curves in each sample were then compared to the control sample in sliding windows of 150 nt with 5 nt steps along the whole genome. The root mean squared deviation of smoothed coverage values between meiotic and control sample in every window was computed after normalization of area under either curve to 1. Novel TSSs and TESs (transcription end sites) are both identified in this analysis as shape difference peaks (Fig 1). Potential TES peaks from the meiotic sample were defined as peaks with a greater average RNA-Seq coverage in the window immediately 5’ of the peak location (with respect to the direction of transcription) than the one immediately to the 3’ side. These peaks were then removed from the analysis. Possible TSS signals were further filtered by the following criteria: (1) the shape difference value at the TSS was required to be larger than median of all shape difference values; (2) the RNA-seq signal was judged to be significant in that the sum of smoothed coverage of meiotic and control samples at the TSS was between the median and 95th percentile; and (3) the difference in signal between the meiotic and control sample at the TSS was required to be larger than 90th percentile. Each TSS was then placed into a contig to define the boundaries of the transcript. Positions both 5’ and 3’ of the TSS were scanned until the difference in expression between the meiotic and control sample was lower than the 90th percentile, defining 5’ and 3’ boundaries of that contig. Contigs of close proximity (those with less than 1 kb between 5’ boundaries) and similar expression levels (less than 3-fold variation of averaged difference of smoothed coverage) were merged. TSS locations were adjusted to be the 5’ end of each contig. Contigs were ranked by a confidence score calculated by multiplying the averaged difference of smoothed coverage and the size of the contig. After contig identification in all three meiotic samples was done, those that were only identified in one sample and those longer than 4 kb were discarded (as really long contigs are likely fusions instead of independent transcripts). The remaining contigs were assigned a new rank by averaging the rankings in all samples in which it was identified, a TSS position based on the average TSS locations and a TSS range based on the variation in TSS locations. Among all of the predicted meiosis specific TSSs, internal TSSs were defined as those with the 5’ boundary of the contig within an ORF and at least 75 nt from the annotated start codon or stop codon, and a contig ranking below 800. Finally, each candidate was visually examined in the genome browser with the raw data, and those likely caused by transcription of an adjacent gene, as well as those with noisy transcription that came through the previous filtering steps were removed (Table 3). Each gene in the refined list was also examined in the tiling array dataset of Lardenois et al., 2010 for indications of internal initiations. Aligned reads and all feature tracks generated from the above analyses were visualized using the Integrative Genomics Viewer v2.3.90 (IGV, https://www.broadinstitute.org/igv/) from the Broad Institute (Cambridge, MA).

**Table 3:** Genes identified to contain mid-sporulation specific internal TSSs.

ORF Name	Gene Name	Start Location^a^	MSE^b^	Lardenois et al.^c^	Gene Description^d^
*YAL043c*	*PTA1*	+1116±145	Yes	Yes	Essential subunit of holo-CPF
*YAR050w*	*FLO1*	+789±20	No	Yes	Lectin-like cell wall protein (flocculin)
***YBL063w***^**e**^	*KIP1*	+497±212	No	No	Kinesin-related motor protein
*YBR069c*	*TAT1*	+585±4	Yes	Ambiguous	Amino acid transporter
***YBR169c***	*SSE2*	+1037±47	No	Ambiguous	Nucleotide exchange factor
*YBR245c*	*ISW1*	+1797±115	No	No	ATPase subunit of chromatin remodelers
***YBR260c***	*RGD1*	+481±152	No	Yes	GTPase-activating protein for Rho3p and Rho4p
***YDL079c***	*MRK1*	+535±11	Yes	Yes	Glycogen synthase kinase 3 homolog; deletion reduces sporulation
***YDL226c***	*GCS1*	+380±34	Yes	Yes	ADP-ribosylation factor GTPase activating protein; deletion abolishes sporulation
***YDR089w***	*VTC5*	+1249±36	No	Yes	Vacuolar transmembrane protein
***YDR143c***	*SAN1*	+479±232	No	No	Ubiquitin-protein ligase
*YDR173c*	*ARG82*	+218±103	No	Ambiguous	Inositol polyphosphate multikinase (IPMK); deletion reduces sporulation
*YDR406w*	*PDR15*	+884±38	Yes	No	Plasma membrane ATP binding cassette transporter
*YDR490c*	*PKH1*	+1256±112	No	Yes	Serine/threonine protein kinase
***YER076c***		+526±92	Yes	No	Protein of unknown function
*YFR005c*	*SAD1*	+671±45	Yes	Yes	Conserved zinc-finger domain protein involved in pre-mRNA splicing; essential
*YGL075c*	*MPS2*	+286±26	Yes	Ambiguous	Essential membrane protein
*YHR082c*	*KSP1*	+584±115	Yes	Yes	Essential serine/threonine protein kinase
*YHR176w*	*FMO1*	+703±139	Yes	Yes	Flavin-containing monooxygenase
*YHR182w*		+1429±62	No	Yes	Protein of unknown function
*YHR211w*	*FLO5*	+2429±7	Yes	Yes	Lectin-like cell wall protein (flocculin)
*YIL078w*	*THS1*	+1647±29	No	Ambiguous	Essential threonyl-tRNA synthetase
*YIL154c*	*IMP2'*	+586±71	Yes	Ambiguous	Transcriptional activator involved in maintenance of ion homeostasis
*YJL092w*	*SRS2*	+915±72	Yes	Yes	DNA helicase and DNA-dependent ATPase; deletion reduces spore viability
*YLL008w*	*DRS1*	+1598±118	No	Yes	Essential nucleolar DEAD-box protein
*YLR106c*	*MDN1*	+11975±97	No	Yes	Huge dynein-related AAA-type ATPase; essential
*YLR273c*	*PIG1*	+706±120	No	Yes	Putative targeting subunit for type-1 protein phosphatase Glc7p
*YLR430w*	*SEN1*	+3696±90	No	Yes	Essential splicing endonuclease
*YML010w*	*SPT5*	+2633±22	No	No	Spt4p/5p transcription elongation factor complex subunit; essential
***YML047c***	*PRM6*	+271±190	No	Yes	Pheromone-regulated membrane protein that transports potassium
***YML072c***	*TCB3*	+1848±8	Yes	Yes	Cortical ER protein involved in ER-plasma membrane tethering
***YNL077w***	*APJ1*	+940±149	No	Ambiguous	Chaperone involved in SUMO-mediated protein degradation
*YNL083w*	*SAL1*	+345±82	Yes	Yes	ADP/ATP transporter
*YNL227c*	*JJJ1*	+1357±54	No	Yes	Co-chaperone that associates with the cytosolic large ribosomal subunit
*YOL057w*		+1283±30	No	Ambiguous	Dipeptidyl-peptidase III
*YOR140w*	*SFL1*	+454±102	Yes	No	Transcriptional repressor and activator
*YOR188w*	*MSB1*	+1443±50	No	Yes	Protein of unknown function
*YOR237w*	*HES1*	+336±132	No	No	Protein implicated in the regulation of ergosterol biosynthesis
*YOR330c*	*MIP1*	+2025±172	No	Ambiguous	Mitochondrial DNA polymerase gamma
*YOR355w*	*GDS1*	+374±85	No	Ambiguous	Protein of unknown function
*YPL174c*	*NIP100*	+1803±13	Yes	Yes	Large subunit of the dynactin complex
*YPL242c*	*IQG1*	+3452±138	No	Yes	Essential IQGAP-related protein
*YPR029c*	*APL4*	+1119±6	No	Ambiguous	Gamma-adaptin; large subunit of the clathrin-associated protein complex

^a^Nucleotide position of the internal TSS relative to the first nucleotide of the annotated ORF, based on the average of TSS locations from at least two mid-meiotic samples. Estimation of initiation range is calculated from the variations in TSS locations.

^b^Denotes whether sequence of 300 nt upstream to the most 3' possible internal TSS position of each gene contains any MSE sites.

^c^Denotes whether similar pattern of transcription is observed in the tiling array dataset from another study [25] (http://sgv.genouest.org/).

^d^Taken from SGD (http://www.yeastgenome.org/).

^e^Shown in bold: ORFs that display internal TSS (localized within the range of start location estimated in the current study) during sporulation in a 5'-end sequencing dataset [10].

To identify potential MSE and URS1 sites upstream of each defined internal TSS, DNA sequences 300 nt upstream to the 3' end of the possible TSS range were examined. Motif analysis was then performed on these sequences using the Motif module from the Biopython libraries (http://biopython.org) with the Position Weight Matrix (PWM) of the Ndt80 or Ime1 binding profile retrieved from ScerTF [50] (http://stormo.wustl.edu/ScerTF/). Sequences 300 nt upstream to each ORF served as negative control. Sites with a PWM score larger than 85% of the possible score range were classified as potential MSE/URS1 sites.

### Analysis for *MRK1* sequence homology

Multiple sequence alignment was performed using the nucleotide sequences of *S*. *cerevisiae MRK1* and homologous regions from *S*. *paradoxus*, *S*. *mikatae*, *S*. *bayanus* and *S*. *kuzriazevii* (GenBank acessions: AABY01000135.1, AABZ01000319.1, AACG02000051.1, JH797022.1) by MAFFT version 7 (http://mafft.cbrc.jp/alignment/server/) with default settings.

### Western blot assays

For western blots, strains A14201 (*MRK1*), ySZ68 (*MRK1*::*3xHA*) and ySZ313 (*mrk1-mMSE*::*3xHA*) were sporulated as described above. 5 ml of cell culture for protein extraction was collected before the switch to SPO medium, at the time of addition of β-estradiol, and two hours after addition of β-estradiol. At these time points, aliquots of cells were fixed in 3.7% formaldehyde, washed, and stained with 4', 6-diamidino-2-phenylindole (DAPI). These samples were examined for progression into the meiotic divisions by epifluorescence with a Zeiss Axioplan 2 microscope. Samples were only processed for western blot experiments if more than 50% of cells had entered the meiotic divisions by two hours after β-estradiol treatment. Cell samples were pelleted, washed, transferred to ice and lysed by alkaline treatment [51]. Pelleted samples were resuspended in SDS sample buffer (10% glycerol, 60 mM Tris-HCl pH 6.8, 0.005% bromophenol blue, 2% β-mercaptoethanol) followed by incubation at 100°C for 5 minutes. Cell debris was removed by centrifugation at 16,000 x g for 3 minutes, and 2 μl of each sample was loaded onto a 10% SDS polyacrylamide gel. Mrk1-3HA was detected using monoclonal anti-HA antibody 12CA5 (BAbCo) at 1:5000 dilution. Porin was detected using monoclonal anti-Por1 antibodies (Molecular Probes) at 1:4000 dilution. HRP-conjugated anti-mouse antibodies (Amersham Biosciences) were used at 1:5000 dilution for detection of Mrk1-3HA and 1:30000 for detection of porin, and proteins were visualized using Clarity-Western ECL substrate (Bio-Rad).

### qPCR

For qPCR experiments, strains A14201 (wild type) and ySZ69 (*mrk1-mMSE*) were sporulated as described above, and 5 ml of culture was collected at the time of addition of β-estradiol and two hours after β-estradiol treatment. Total mRNA was extracted and purified with a RiboPure Yeast Kit (Ambion). cDNA was synthesized from the mRNA using the qScipt cDNA SuperMix (Quanta Bioscience) at a final RNA concentration of 50 ng/μl, and then treated with RNase A (0.5 μg/μl) at 37°C for 30 minutes. The cDNA reaction mix was diluted 10-fold, and 3 μl was used in each 10 μl reaction with LightCycler 480 DNA Sybr Green I PCR master mix (Roche) to determine the mRNA level of each gene using a Mastercycler EP Realplex (Eppendorf). Each sample was assayed in triplicate in each experiment, and all experiments were performed at least twice.

### Ether sensitivity assay

To test for ether resistance, cells were sporulated as described above, and sonicated briefly to break the clumps into separate cells. For each sample, 100 cells were counted for sporulation efficiency. Cell concentration was measured with a Z1 Beckman-Coulter Counter, and with this information, each culture was diluted so that the final concentration of asci was 5x10^6^ asci/ml, and 5-fold serial dilutions were spotted identically on two YPD plates. One plate served as a no-ether control, while the other was inverted over ether soaked filter paper (Whatman #3, 1003–090) for 1 hour. Plates were incubated at 30°C for 2–3 days and scanned.

## Results

### A bioinformatic pipeline for detection of meiosis-induced internal transcription start sites

Transcriptomic datasets, e.g. microarray and RNA-Seq data, can enable observations of changes in TSSs over a meiotic time-course [2, 10, 25]. We analyzed an RNA-Seq dataset of a highly synchronized meiotic time-course (GSE90008). The culture was synchronized by first arresting the cells in sporulation media and then inducing *IME1* and *IME4*, two important regulators of meiotic entry (Yifeng Xu, personal communication). This system produces highly synchronized entry into meiosis enabling efficient sporulation at fine time-resolution [47, 52]. The cDNA library in the study was prepared with random hexamer primers, which is preferential to oligo(dT) primed libraries that generate bias towards 3' ends [53]. In order to capture novel TSSs regardless of their positions relative to genomic features, we developed a customized bioinformatic pipeline. The work flow of analysis is shown in Fig 1.

After mapping and quantifying the RNA-Seq reads from each sample to the genome, each meiotic sample was compared to the control to look for TSSs specific to the meiotic sample. To reduce fluctuations of raw read pileups between adjacent genomic positions, the histogram of sequence reads was first smoothed across the genome. Then, these smoothed coverage profiles were compared between the control and each meiotic sample. Differences in the shape of the transcription profiles were identified computationally as Root Mean Squared Deviation (RMSD) of normalized coverage values between the samples in sliding windows. Locations of shape difference peaks were then filtered to remove potential TES signals and weak TSS signals.

Because of the high sensitivity in RMSD-based detection for shape differences, TSS signals were called not only in positions representing true TSSs, but also from locations where the expression profile appeared wavy due to noise in the data. To distinguish between the two situations, for each TSS peak, a transcript contig was constructed, the boundaries of which were defined from the difference in smoothed coverage between the meiotic sample and control. Nearby contigs of similar expression levels were merged, and the final TSS predictions were defined to be the 5'-end of the contig containing that TSS. To rank the contigs by confidence level, each contig was assigned a confidence score based on two parameters: (1) expression level of the contig; (2) size of the contig, as longer contigs are more likely to represent true novel transcripts than shorter ones, which tend to result from noise. Three time-points taken from cells after the induction of *NDT80* (3.75 h, 4 h, 4.25 h after addition of copper) were processed to identify contigs, and only contigs identified in at least two samples were kept. A final ranking was assigned to each contig by averaging all rankings of that contig in different samples. Similarly, the final TSS location was assigned by averaging all TSS locations in different samples, with a range estimate that reflects variation in TSS locations.

To identify internal initiation events, each transcript contig was evaluated for whether its TSS lies internal to an annotated ORF. In the top 800 ranked contigs, 100 were identified to have TSSs internal to the coding region. The corresponding ORFs were then visually examined in the original mapping profile to exclude cases where the internal initiation likely represents transcription from an adjacent gene or noisy data that came through the informatics filters. Further details, including parameters used for the analysis, are described in Material and Methods. A description of all sporulation-specific contigs is available in S1 File.

In sum, this approach identified 43 genes that display potential internal TSSs after *NDT80* induction (Table 3). Visual examination of transcription data for these genes in a previously published tiling array dataset (http://sgv.genouest.org, [25]]) confirmed internal initiations of 24 of the 43, suggesting the robustness of this pipeline. From a 5'-end sequencing dataset which attempted to capture budding yeast TSSs at a genome-wide scale [10], direct evidence was found for 11 genes on our list to have meiosis specific TSS at similar positions, supporting the idea that the 5'-boundary of these transcript contigs corresponds to true initiation sites.

### Mid-sporulation specific internal initiations are *NDT80* dependent

This analysis identified many potential TSSs in genes transcribed after *NDT80* induction. However, whether these alternative initiations are coupled to *NDT80* induction or arise earlier in meiosis and persist after *NDT80* induction is not clear. To address this question, we applied the same pipeline to early meiotic samples from the same time-course. The three early meiotic samples we chose to analyze (0.25 h, 0.5 h and 0.75 h after addition of copper) represent the time immediately after *IME1* and *IME4* are induced, when well-studied early meiotic genes like *IME1*, *IME2* and *RIM4* are highly expressed. This analysis revealed a similar number of genes displaying potential internal TSSs (S2 File). However, only two genes (*MPS2*, *YOL057w*) were identified in both the early and mid-meiotic samples. This suggests that while internal initiations are not unique to a particusslar time in meiosis the specific internal TSSs listed in Table 3 may largely be induced by Ndt80 activity.

To examine the likelihood that these internal initiations are driven by Ndt80, sequences upstream of the predicted start sites were examined. Most regulatory motifs lie within 300 bp upstream of the TSS and this is also true for MSE sites in *NDT80*-induced genes [54, 55]. Therefore, 300 nt of sequence upstream to the 3'-end of the predicted range of TSS position for each internal initiation was examined for MSE sites using a Position Weight Matrix (PWM) for the Ndt80 binding site (see Materials and Methods). The upstream regions of 17 mid-meiotic internal TSS were found to contain MSE sites (Table 3), an extremely significant enrichment (p<1.5x10^-22^, Fisher's exact test). When the same analysis was used to look for binding sites for the early meiotic transcription factor Ime1, no enrichment for URS1 sites was found in these sequences. Also, no enrichment was found for either MSE or URS1 sites in the upstream regions of early-meiotic internal TSSs. These results are consistent with the idea that induction of *NDT80* results in internal transcription initiation in several of its target genes.

Internal initiation and *NDT80*-dependence was tested by quantitative PCR (qPCR). Synchronous progression into the meiotic divisions can be achieved by placing the *NDT80* gene under the control of an estradiol-inducible promoter (*NDT80-IN*) [45, 46]. Cells homozygous for this allele arrest in meiotic prophase due to the absence of *NDT80* expression and then proceed synchronously into the meiotic divisions upon addition of estradiol to the culture. A strain homozygous for *NDT80-IN* was cultured in sporulation medium for 6 hours, allowing the cells to progress to the *ndt80*Δ arrest point and then estradiol was added to induce *NDT80* transcription. Four candidate genes; *MRK1*, *IQG1*, *DRS1* and *SAD1* were selected for analysis because all appear to have internally initiated transcripts after *NDT80* expression in our analysis and in multiple previous datasets [2, 10, 25]. Moreover, in a ribosome profiling dataset, all four display increased ribosome binding near the predicted internal TSS after *NDT80* expression [3]. *CDC3* and *CDC10* were included as controls since both genes are known to be up-regulated following *NDT80* expression, but do not display internal start sites in any dataset examined [1, 2, 10, 25, 56].

**Fig 2:**
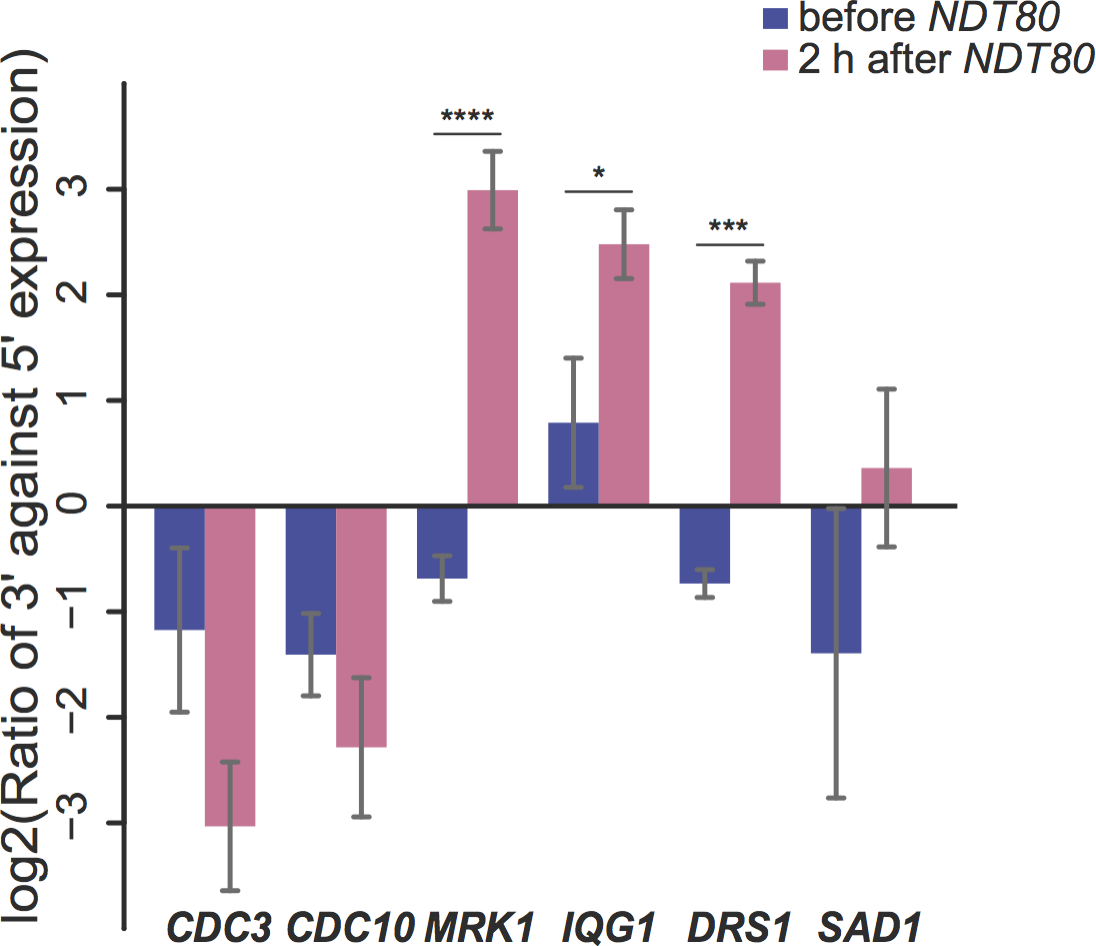
qPCR examination of *NDT80* dependent 3' transcriptional induction for representative genes with mid-sporulation specific internal TSS. Wild-type (A14201) strain was synchronized in sporulation using β-estradiol inducible *NDT80*. The levels of 3' and 5' expression of control transcripts (*CDC3* and *CDC10*) and transcripts of a subset of genes identified with mid-sporulation specific internal TSSs (*MRK1*, *IQG1*, *DRS1* and *SAD1*) were measured by qPCR of samples harvested when β-estradiol was added to the media and 2 hours after *NDT80* induction. The ratios of 3' expression to 5' expression are shown on a log2 scale. Values shown are the averages from two or more independent experiments. Error bars represent standard error of the mean. Statistical differences were examined by Student's t-test. Symbols denote statistical significance (*  =  p<0.05; ***  =  p<0.001; **** =  p<0.0001).

For each gene, two pairs of primers were used, one pair in the portion of the gene 5’ of the internal start site and one pair that amplified from the 3’ side, within the internally initiated transcript. The presence or absence of transcriptional induction internal to the ORF (i.e. whether more expression is detected from the 3’-end than the 5’-end of the ORF) was determined by taking the log ratio of the level of expression in the 3’ primer pair relative to the 5' pair (log_2_[(3' expression)/(5' expression)]) so positive values indicate increased expression of the 3' end of the ORF. Unlike *CDC3* and *CDC10* where both genes display more transcription in the 5' portion of the gene, all 4 genes tested show 3' transcriptional induction specifically after *NDT80* induction (Fig 2). These inductions are significant (Student's t-test; p<5.54×10^−7^ for *MRK1*, 0.03 for *IQG1*, 3.02×10^−4^ for *DRS1*) for all except *SAD1* (p<0.32). These qPCR results confirm our bioinformatics analysis for three out of four genes and suggest that Ndt80 is directly responsible for the internally initiated transcripts.

### Internal initiation expands potential proteome diversity during sporulation

**Fig 3:**
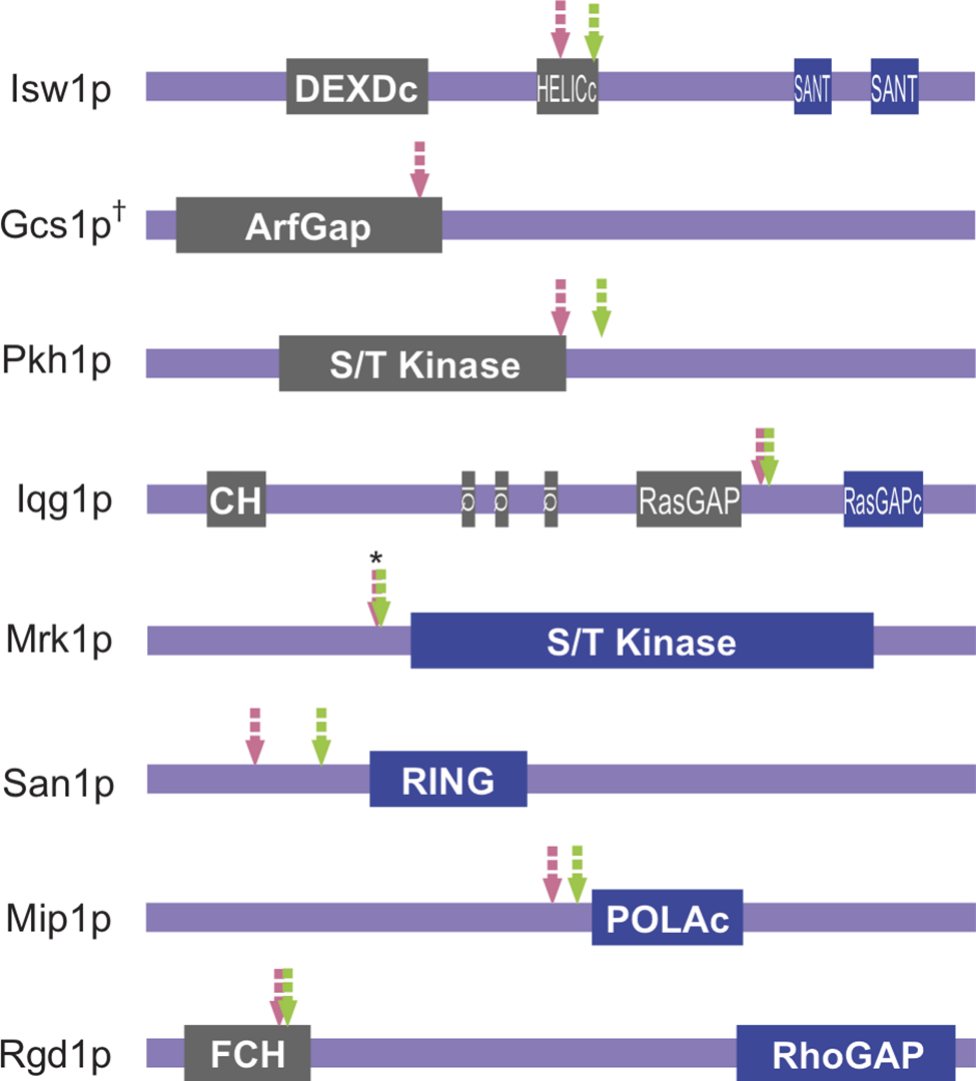
Protein domain structure of example targets from the analysis. Schematic diagram showing domain organizations of gene products of 8 candidates from Table 3. Predicted motifs from Pfam (http://pfam.xfam.org/) and SMART (http://smart.embl-heidelberg.de/) are illustrated on each protein. Pink arrows denote the amino-acid position corresponding to the 5’ most position of the range for each internal TSS. Green arrows refer to the first in-frame methionine encoded by each internally initiated transcript. Motifs C-terminus to each arrow are shown in blue, while those to the N-terminus are shown in grey. Asterisk (*): for Mrk1p, since both the predicted TSS location and the first methionine encoded by its internal TSS are located inside the intron (Fig 4A), both features are marked at the exon 1 and 2 junction. †: No in-frame start codon can be found 3’ to the predicted internal TSS of *GCS1*.

The internally initiated transcripts potentially encode N-terminally truncated proteins. These shorter isoforms of the proteins could have distinct function or regulation from the full-length protein. The predicted domain structure of each protein was examined with respect to the position of the internal TSS and the first in-frame ATG codon. Several examples are shown in Fig 3. In some cases, the shorter transcript encodes a predicted catalytic domain separate from regulatory sequences, for example the GAP domains of Rgd1 and Iqg1 or the kinase domain of Mrk1. Conversely, other transcripts would express regulatory regions without catalytic activity, such as the SANT domains of Isw1 or the C-terminal region of Pkh1. These examples show the potential of internal initiations to expand the diversity of the meiotic proteome.

### An MSE site within the intron is required for the expression of the internally initiated *MRK1*

**Fig 4:**
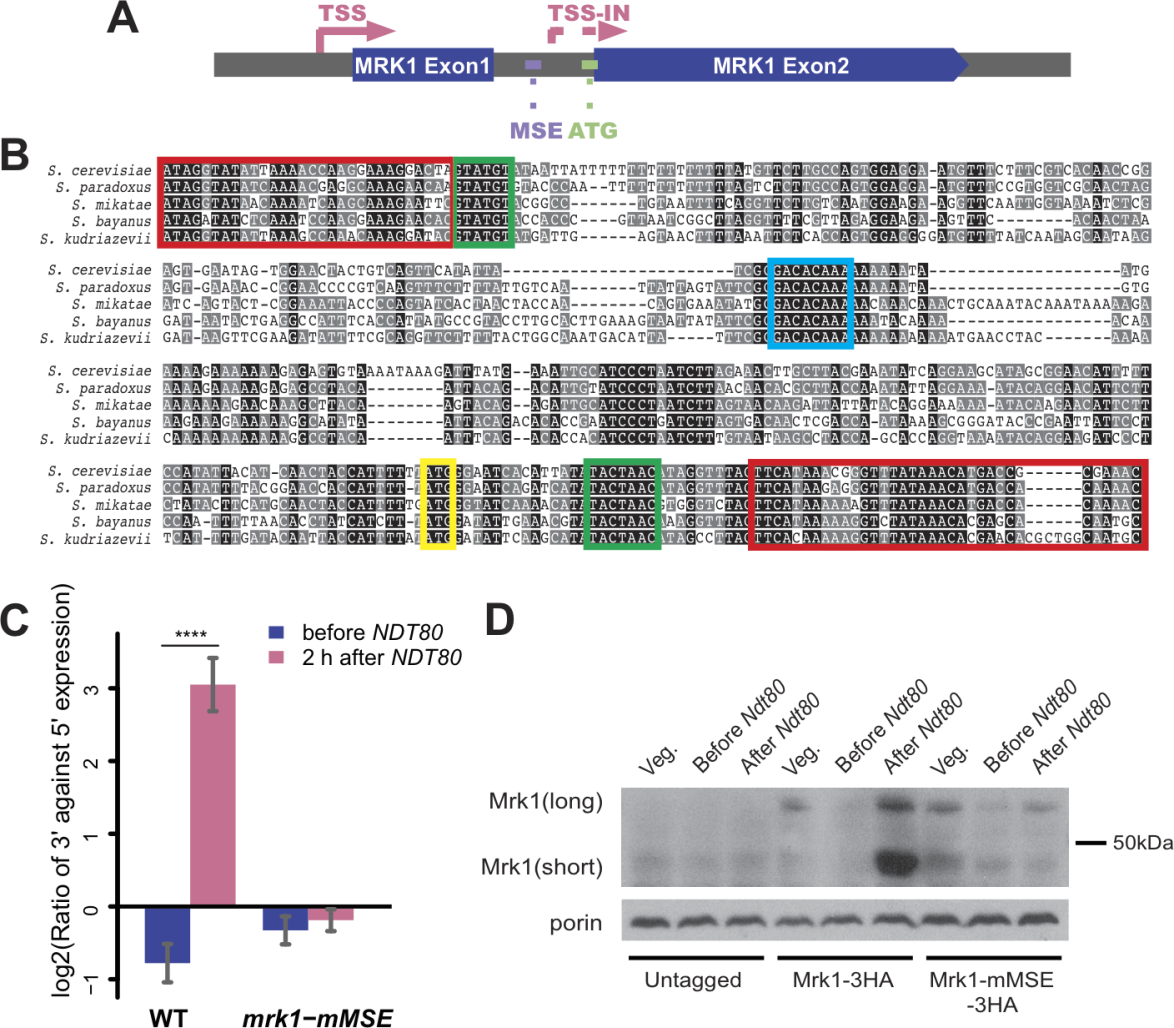
Induction of internally initiated *MRK1* is regulated by Ndt80. **(A) *MRK1* gene structure.** Exons are indicated in blue boxes. Solid arrow: TSS upstream of the ORF defined by 5'-end sequencing [10]. Dashed arrow: internal TSS (TSS-in) defined in this study. Purple dot: location of MSE motif in the intron of *MRK1*. Green dot: location of the first in-frame start codon of the internally initiated transcript. **(B) Multiple sequence alignment of *MRK1* intronic region from *S*. *cerevisiae* and the homologous regions of closely related species.** Conservation of residues is indicated by shading: black- identical; grey- >60% similarity. Red boxes indicate exon sequences. Green boxes indicate the 5’ splice site and branchpoint consensus sequences. Cyan box indicates core MSE consensus and yellow box denotes the predicted start codon of the internally initiated *MRK1*. **(C) qPCR examination of *NDT80* dependent 3' transcriptional induction of *MRK1* in wild-type and MSE mutant.** Wild-type (A14201) strain and *mrk1-mMSE* strain (ySZ69) were synchronized in sporulation using β-estradiol inducible *NDT80*. The levels of 3' and 5' expression of *MRK1* were measured by qPCR of samples harvested when β-estradiol was added to the media and 2 hours after *NDT80* induction. The ratios of 3' expression to 5' expression are shown on a log2 scale. Values shown are the averages from 7 or more independent experiments. Error bars represent standard error of the mean. Statistical differences were examined by Student's t-test. Symbols denote statistical significance (**** =  p<0.0001). **(D) Detection of Mrk1 isoforms by western blot analysis.** Wild-type (A14201), *MRK1-3HA* (ySZ68), and *mrk1-mMSE-3HA* (ySZ313) strains were synchronized in sporulation using β-estradiol inducible *NDT80*. For each strain, cells grown in YPA, when β-estradiol was added to sporulation media and 2 hours after *NDT80* induction were analyzed.

Among the genes we examined with qPCR, *MRK1* has the strongest induction of the internally initiated transcript, with as high as 8-fold higher expression of the 3' end relative to the to 5' region (Fig 2). *MRK1* is the only one of the four GSK-3β homologs in yeast that contain an intron ([57]], Fig 4A). The first exon encodes an extra N-terminal extension unique to Mrk1p compared with the other members of the GSK-3 family in budding yeast, while the kinase domain is contained entirely within the second exon. A consensus MSE site is found within the intron 5’ to the internal TSS. Also within the intron, 3’ of the TSS lies an ATG codon that is in frame with the second exon of *MRK1*. Translation beginning at this ATG would produce a protein isoform consisting of 11 amino acids encoded within intron sequences plus the entire protein kinase domain encoded by the second exon (Fig 4A). The positioning of these sequence elements strongly suggests that the internally initiated transcript of *MRK1* is directly regulated by Ndt80 at the MSE site, and translated into a shorter protein.

The intron in *MRK1* is only found in closely related *Saccharomyces* species. To investigate the conservation the MSE of we aligned the nucleotide sequences of the *MRK1* intron in five *Saccharomyces* species (Fig 4B). Sequences within the intron are divergent in the different species, but both the MSE site and the predicted ATG codon of the internally initiated *MRK1* are perfectly conserved. Taken together, the sequence analysis suggests that in the *Saccharomyces* cluster, the MSE site in the *MRK1* intron serves as a functional element preserved during evolution.

To test the idea of direct *NDT80* regulation, CRISPR/CAS9 was used to mutate the MSE site in the intron in the native chromosomal context. A diploid homozygous for this mutation (*mrk1-mMSE*) in the *NDT80-IN* background was generated and internal initiation was examined by qPCR before and after *NDT80* induction. Contrary to wild-type, the *mrk1-mMSE* allele did not display increased 3’ transcription after *NDT80* was induced (Fig 4C). To examine whether these changes in transcription ultimately affect protein expression, three tandem copies of the hemagglutinin (HA) epitope were inserted at the 3’ end of the coding sequence of both *MRK1* and *mrk1-mMSE* and protein levels were monitored by Western blot with anti-HA antibodies. Whole cell lysates were made from cells prior to transfer to SPO medium, at the time of estradiol addition, and 2 h after induction of *NDT80* by addition of estradiol. In the *MRK1-HA* strain, full-length Mrk1 was observed in all samples, with a lower amount in pre-*NDT80* meiotic sample and somewhat elevated expression in post-*NDT80* meiotic sample (Fig 4D). A faster migrating band, corresponding to the predicted size of the N-terminally truncated Mrk1 expressed from the internally initiated transcript was seen only in the post-*NDT80* meiotic sample. In contrast, the *mrk1-mMSE* strain exhibited similar levels to wild-type of the full-length protein across growth conditions, but no expression of the short protein was observed after *NDT80* induction. In sum, Ndt80 likely directly regulates internal initiation of *MRK1* by binding to the MSE site present in the intron and this results in the translation of an N-terminally truncated form of Mrk1 in meiotic cells.

### Internally initiated *MRK1* promotes sporulation redundantly with *RIM11*

**Fig 5:**
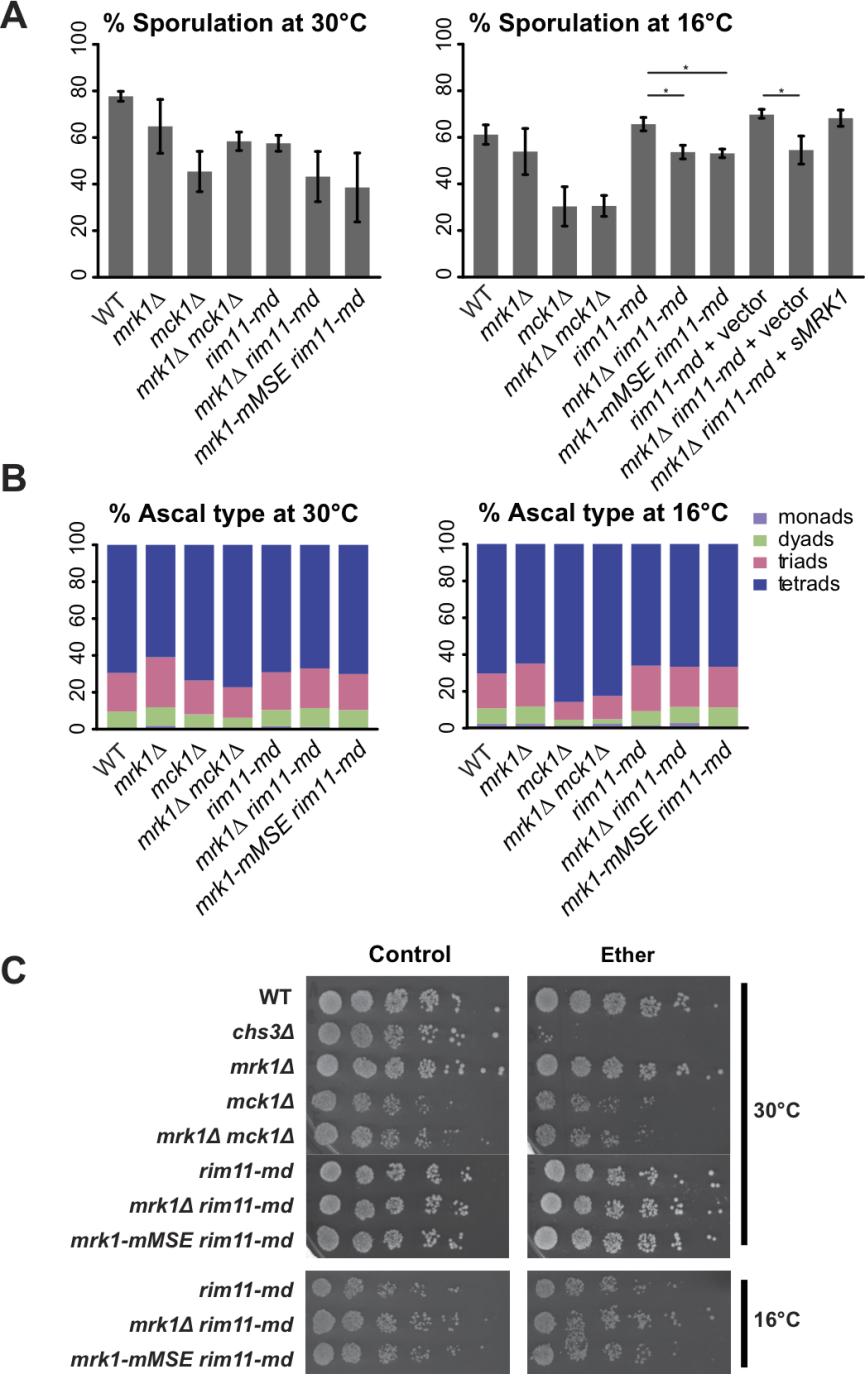
The effect of *MRK1* deletion or depletion of internally initiated *MRK1* on sporulation and spore wall integrity. Strains of indicated genotypes were sporulated in liquid (WT-AN120; *chs3*Δ-AN262; *mrk1*Δ-ySZ102; *mck1*Δ-ySZ23; *mrk1*Δ *mck1*Δ-ySZ46; *rim11-md*-ySZ39; *mrk1*Δ *rim11-md*-ySZ135; *mrk1-mMSE rim11-md*-ySZ98; *rim11-md*+vector-ySZ317; *mrk1*Δ *rim11-md*+vector-ySZ318; *mrk1*Δ *rim11-md*+*sMRK1*-ySZ315). Cells were allowed to sporulate either at 30°C for >48 h, or at 16°C for >96 h. **(A) Sporulation efficiency.** At least 300 cells of each strain were counted in each experiment. Bar plots represent the mean sporulation efficiency of at least 3 independent experiments. Error bars represent standard error of the mean. Statistical differences of strains in *rim11-md* background were examined by Student's t-test. Symbols denote statistical significance (* =  p<0.05). **(B) Ascal type distribution.** At least 125 ascus of each strain were counted in each experiment. Bar plots represent the mean ascal type distribution of at least 3 independent experiments. **(C) Ether resistance of spores.** Each sporulation culture was diluted to a concentration of 10^6^ asci/ml, and 5-fold serial dilutions were spotted onto YPD plates. Left panels, plates unexposed to ether. Right panels, plates exposed to ether vapor for 30 minutes.

In a systematic screen of the *S*. *cerevisiae* knockout collection, deletion of *MRK1* was found to moderately reduce sporulation efficiency [58]. However, no effect on sporulation was reported in two other genome-wide screens [59, 60]. To re-examine the effect of *MRK1* knock-out on sporulation efficiency, we created a homozygous deletion of *MRK1* in the SK1 strain background. Mean sporulation efficiency of *mrk1*Δ appeared 13% lower than that of wild-type, although the difference is not statistically significant (Fig 5A, left panel). Thus, *MRK1* is not important for sporulation under these conditions.

The *S*. *cerevisiae* GSK-3 homologs have been shown to have overlapping functions [28, 33], and both *RIM11* and *MCK1* have been shown to have roles in meiosis [28, 35]. The lack of sporulation phenotype of *mrk1*Δ could be related to redundancy with *RIM11* or *MCK1*. Deletion of *MCK1* reduced sporulation efficiency relative to wild-type, consistent with earlier reports [29]; however, deletion of *MRK1* did not exacerbate the *mck1*Δ phenotype (Fig 5A, left panel). This result suggests that *MRK1* does not act redundantly with *MCK1* in sporulation.

Deletion of *RIM11* blocks entry into meiosis [35]. This defect occurs long before the induction of *NDT80* and could, therefore, mask functions of *RIM11* later in the meiotic program. To bypass this early role of *RIM11* a meiotic depletion allele, *rim11-md*, was constructed, in which *RIM11* expression is under the control of the meiotically inactive *CLB2* promoter [43]. A diploid hemizygous for this allele (*rim11-md*/*rim11*Δ) sporulated well, indicating that one *rim11-md* allele provides sufficient protein to fulfill Rim11 function early in sporulation. Deletion of *MRK1* in the *rim11-md*/*rim11*Δ background caused a reproducible, but not statistically significant (p<0.16, Student’s t-test) reduction in sporulation efficiency (Fig 5A, left panel). In vegetative cells, *mck1*Δ phenotypes are exacerbated by cold and high-temperature [61]. Sporulation was therefore examined in this same set of strains with incubation at lower (16°C) and higher (37°C) temperatures (Fig 5A, right panel). While no differences were seen at higher temperature (unpublished observations), at lower temperature a modest but significant reduction in sporulation was seen when *mrk1*Δ was combined with *rim11-md* (p<0.036 for *mrk1*Δ in *rim11-md*; p<0.040 for *mrk1*Δ in *rim11-md* carrying the vector plasmid). This same effect was seen when the *mrk1-mMSE* allele was present rather than *mrk1*Δ (p<0.021). The reduction could be rescued by ectopic expression of the internally initiated form of *MRK1* alone (*mrk1*Δ *rim11-md*+*sMRK*). This suggests that *MRK1* acts in concert with *RIM11* to promote spore formation and that it is specifically the truncated form of *MRK1* induced by *NDT80* that is responsible.

Mutants that only modestly reduce sporulation efficiency, defined as the fraction of cells that produce asci, can have stronger effects when the number of spores present in each ascus is considered. For example, modest defects in meiotic chromosome segregation or meiotic spindle pole body function do not inhibit sporulation but shift the major ascal type from tetrads to dyads [62, 63]. For each of the strains bearing mutations in different GSK-3 family genes, the number of spores contained in the asci was assessed. No strong effect on the distribution of ascal types was seen for any of the mutants, regardless of the temperature at which sporulation occurred (Fig 5B).

*NDT80* induces the expression of many genes important for spore wall assembly and mutation of genes in this class can result in defects in spore wall function without changes in the ability of the cell to produce visible spores [6]. To evaluate spore wall functionality, the viability of the spores after exposure to ether vapor, a common assay for spore wall integrity [64], was examined (see Materials and Methods). Serial dilutions of sporulated cultures were spotted on to rich media and exposed to ether vapor before incubation to allow germination and growth. A *chs3*Δ strain was used as a control for ether sensitivity ([64, 65], Fig 5C). We did not observe notable defects in ether resistance in either *mrk1*Δ or *mrk1-mMSE* (Fig 5C). Therefore, after examining all these aspects of sporulation, we conclude that the internally initiated *MRK1* has redundant functions to *RIM11* in regulating the efficiency of sporulation.

## Discussion

Earlier studies have reported widespread changes in transcript initiation sites during sporulation [2, 10, 25]. Here we report an informatics analysis of RNA-Seq data to identify TSSs that occur within annotated coding regions during budding yeast meiosis. Dozens of genes are identified to have internal TSSs specific to sporulation, suggesting that internal transcription initiation is not a rare phenomenon. A previous RNA tiling array study also suggests sporulation-specific internal initiation for about half the genes identified and the presence of these shortened transcripts was confirmed by qPCR for several of the genes. Moreover, at least 11 of these genes have been found to display 5'-cap structures proximal to the predicted start locations in sporulating cells [10]. Thus, our analysis is able to efficiently and correctly identify *bona fide* TSSs.

Early and mid-sporulation transcriptomes reveal distinct sets of genes with internal TSSs. The dynamic switch of internal TSSs along this developmental pathway suggests a tightly regulated process. For those internal TSSs appearing in mid-sporulation, MSE sites are significantly enriched in the sequences upstream to these internal TSSs. Further, qPCR analysis of four genes that have MSE sites 5' to the internal TSSs demonstrates that Ndt80 leads to internal transcription of *MRK1*, *IQG1* and *DRS1*, and mutation of an MSE site within the *MRK1* intron abolished the expression of the internal transcript. Thus, the appearance of these internal TSSs is likely driven directly by Ndt80 as part of the general induction of mid-meiotic genes. *NDT80* is expressed only during sporulation, so these shorter transcripts and the truncated proteins they encode are sporulation specific. Placing enhancer binding sites within the coding region may be a common mechanism to increase protein diversity during developmental processes.

How does internal initiation affect which protein variant is expressed, and how does this impact sporulation? Of 43 genes with mid-sporulation specific internal initiations, only three genes—*MRK1*, *GCS1* and *SRS2*, have been previously associated with roles in sporulation or germination [58, 66, 67]. There are a number of reasons earlier studies may not have revealed sporulation phenotypes for mutations in these genes. First, 10 genes on the list out of the 43 are essential and so their possible role in sporulation cannot be assessed by deletion alleles. A second possibility is that the shorter proteins expressed from the internal transcripts may act to inhibit the function of the full-length protein. From the analysis of protein domain structures, the genes fall into two general categories. In the first instance, the predicted truncated proteins contain only a catalytic domain, such as Rgd1, Mrk1, San1 and Mip1. In the second instance, the shorter transcripts encode potential regulatory domains lacking a catalytic domain, such as Isw1, Gcs1, Pkh1 and Iqg1 (Fig 3). For the latter class, if the full-length protein itself impairs sporulation, the shorter proteins produced by internal initiations could counteract that effect. In this circumstance, deletion studies would eliminate both forms of the protein and have no effect on sporulation efficiency. As with the internal initiations that occur in essential genes, it will be necessary to identify mutations that eliminate the internal TSS without affecting expression of the intact gene in order to test the significance of each transcript for sporulation efficiency.

Redundant gene functions might also account for why no sporulation phenotypes have been reported for deletions in some of the genes with internal TSSs. Redundant functions are common amongst genes in the *NDT80*-regulon [68], and overlap between *MRK1* and *RIM11* appears to account for the lack of phenotype of the loss of *MRK1*. The sporulation defect seen in the *mrk1*Δ *rim11-md* diploids is quite mild; however, this might reflect insufficient depletion of the Rim11 kinase rather than a minor role for these kinases in sporulation. While the *CLB2* promoter is an effective means to shut off expression, whether complete depletion is achieved depends on the stability of the mitotically expressed protein and how much protein is required for activity. An alternative strategy to inactivate the kinase is to engineer mutations into active site that render it sensitive to specific ATP analog inhibitors [69]. This has been used to acutely inhibit kinases during sporulation and can be used to separate functions of a kinase that occur early and late in the process [45, 70, 71]. Such “analog-sensitive” alleles of *RIM11* were constructed, but were not effectively inhibited by the available analogs (S.Z., unpublished observations). Nonetheless, future development of an effective Rim11 shutoff system could aid in defining the functions of *MRK1* and *RIM11* late in sporulation.

It is also possible that some of the genes with internal initiations have no sporulation phenotype when deleted because the internal initiations represent transcriptional ‘noise’. That is, transcription triggered by random Ndt80 binding sites that are simply neutral and do not have an evolutionarily selected function. The internal initiation of *MRK1*, however, appears to be positively selected in a cluster of *Saccharomyces* species, as the intronic MSE site is much more conserved in a highly variable genomic context. Nonetheless, the proportion of functionally important internal initiations remains unclear.

The prevalence and dynamic properties of internal transcriptional initiation observed are unlikely to be specific to budding yeast sporulation. Alteration in TSSs involving internal initiations have also been observed in budding yeast under different growth conditions, when fission yeast undergo meiosis, during Drosophila embryonic development and in human cells of different tissue types and of disease states [17, 72–74]. The analysis pipeline described performs a comparison of transcript architecture on RNA-Seq data independent of genome annotations, and thus can be adapted to other RNA-Seq datasets in other systems for novel TSS detection. Such investigations could unveil the prevalence, regulation and functional importance of internal transcriptional initiation in other processes.

## Supporting information

S1 File All novel transcript contigs identified from early and mid-meiotic samples. (XLSX)

S2 File Genes identified to contain early-sporulation specific internal TSSs. (XLSX)

S3 File Python script used for the analysis of novel internal TSS identification. (TXT)
